# The Diagnosis and Treatment of Multiple Factitious Oral Ulcers in a 6-Year-Old Boy

**DOI:** 10.1155/2017/1986834

**Published:** 2017-02-15

**Authors:** Priscilla Santana Pinto Gonçalves, Daniela Alejandra Cusicanqui Mendez, Paulo Sérgio da Silva Santos, José Humberto Damante, Daniela Rios, Thiago Cruvinel

**Affiliations:** ^1^Department of Pediatric Dentistry, Orthodontics and Public Health, Bauru School of Dentistry, University of São Paulo, Bauru, SP, Brazil; ^2^Department of Surgery, Stomatology, Pathology and Radiology, Bauru School of Dentistry, University of São Paulo, Bauru, SP, Brazil

## Abstract

Factitious ulcers are characterized by self-inflicted lesions with multifactorial origin. These lesions are frequently found in head, neck, and hands. This report shows a 6-year-old boy diagnosed with factitious oral ulcers that occurred after the self-biting of buccal vestibule and nail-scratching of gingival tissue. Clinically, a significant swelling was observed, hard on palpation, located at the right lower third of the face, next to the posterior area of the mandible. In the intraoral examination, ulcers at different healing stages were noted on the swelling area. During the anamnesis, the father reported a change in his familial structure that triggers psychological stress, providing the clues to the presumptive diagnosis of factitious oral ulcers. We prescribed the topical use of Gingilone® three times a day to control the local pain and inflammation. At 7-day follow-up, we noticed the reduction of extraoral swelling and the initial healing of the ulcers. The presumptive diagnosis was confirmed at 30-day follow-up, with the lasting remission of oral lesions. The treatments of factitious oral ulcers should be individually tailored for each patient, focused on a multidisciplinary approach, including psychotherapy and periodic clinical control. To the best of our knowledge, gaps of evidence lead to the lack of standardized clinical protocols on this issue.

## 1. Introduction

The factitious ulcers are characterized by self-inflicted lesions related to multifactorial origin, such as accidents and/or chronic habits [1]. Such lesions are frequently located on head, neck, and hands [2]; the dentists can easily detect factitious oral ulcers in their daily clinical routine [1, 3].

People more susceptible to develop factitious ulcer include individuals with syndromes, insensitivity to congenital pain, intellectual disability, autism, or schizophrenia, besides children from unstructured families, homeless people, drug addicts, and sexual abuse victims [2, 4, 5]. The individuals with emotional or psychological disturbances may develop masochistic attitudes towards self-harm [1, 3, 4], with the purpose of seeking their family attention [4].

The factitious ulcers in nonsyndromic individuals are either intentional (functional) or unintentional (organic) [2]. The intentional self-harm occurs in individuals with psychological/emotional disturbances who seek help or attention [2]. The unintentional self-harm appears during stressful situations related to chronic deleterious habits, such as bruxism, thumb, or lip sucking, and the* morsicatio buccarum *[6]. These habits begin from an initial accidental injury that continues to damage, not allowing the natural healing of lesion [6, 7].

The prevalence of factitious ulcers among nonsyndromic individuals seems to be higher among children, teenagers, and young people (17 to 38%) compared to adults (4%) [2]. Also, females are more prone to have self-harm episodes [8]. The ulcers are located in any anatomic structure of oral cavity [6]. Nevertheless, the lower lip and the tongue are most affected by biting [2]. The treatments vary according to the lesion severity and the frequency of the self-harm act, being based on psychological, pharmacological, and physical restrain and, in severe cases, dental extraction of the teeth near the injury [9].

This report shows a singular clinical case of factitious oral ulcers in a 6-year-old boy without systemic disease.

## 2. Case Presentation

A 6-year-old boy accompanied by his father came to the Clinics of Pediatric Dentistry and Stomatology of the Bauru School of Dentistry, University of São Paulo (FOB-USP). The father previously signed the free consent form, authorizing the treatment and the publication of this case. During the anamnesis, the father reported that they first sought a doctor suspecting of parotiditis that was not confirmed. The doctor referred the child to the dentist. Then, the father reported his concern with an abnormal tumescence that appeared in the right side of the boy's face two days ago. This lesion was associated with pain on palpation. Other relevant clinical findings were the allergy to insect bites and tonsillectomy performed eight months ago. The boy was not under medical treatment or medication use.

**Figure 1:**
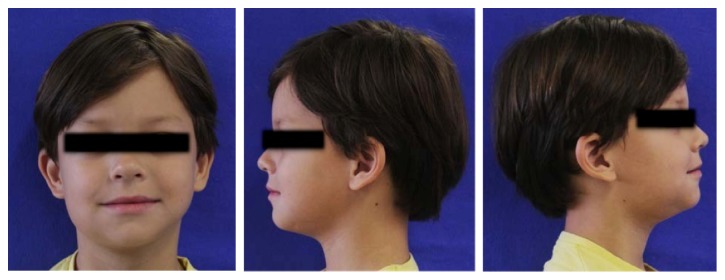
The initial clinical aspect of the child. Note the presence of swelling on the right lower third of the face.

**Figure 2:**
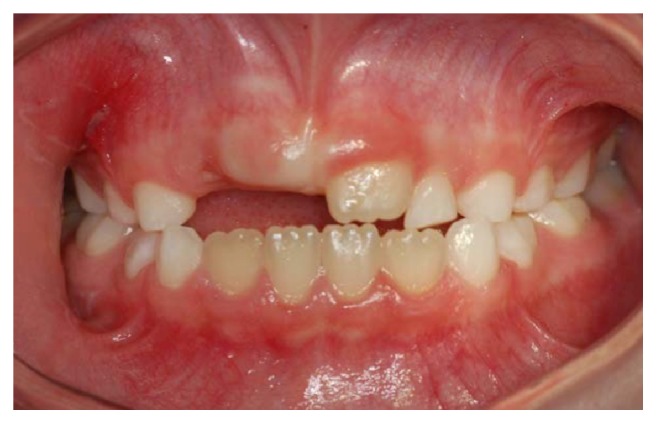
Frontal clinical view of the maxillary and mandibular arches.

**Figure 3:**
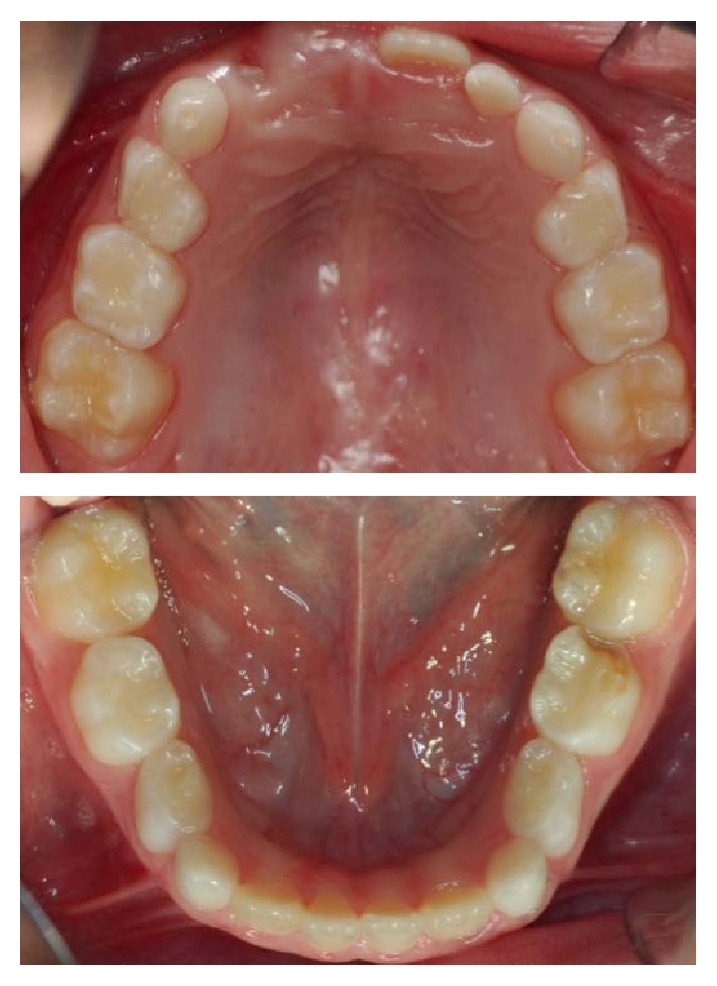
Maxillary and mandibular occlusal views. Note the presence of a dental caries lesion developed on the hypomineralized distal-occlusal surface of tooth #75. There is no evidence of clinical alterations in the right mandibular teeth and mucosa.

**Figure 4:**
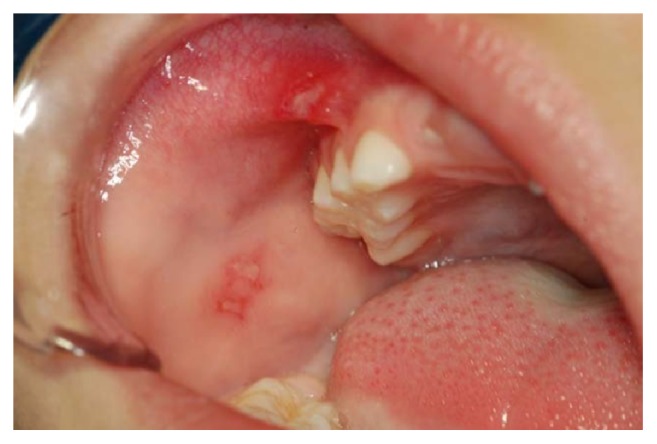
The initial clinical aspect of the ulcerated mucosa. Note the presence of factitious oral ulcers and localized swelling. The lesions were produced by self-biting of buccal vestibule and nail-scratching of gingival tissue.

The extraoral clinical examination revealed that the patient had a significant swelling, hard on palpation, in the right lower third of the face, next to the posterior area of the mandible's body (Figure 1). In the intraoral examination, we noted a dental caries lesion on the left second primary molar (#75). Notwithstanding, either other alterations in hard and soft tissues or premature occlusal contacts were not found at the right side of the mandibular arch (Figures 2 and 3). The oral mucosa located on the swelling area had ulcers at different healing stages (Figure 4). The boy affirmed that he did not remember when the lesions had started, reporting pain only on palpation. The analysis of an orthopantomography confirmed no bone or tooth alterations that could justify the swelling.

During the session, the boy was very quiet and cooperative, demonstrating his shy behavior. In the anamnesis, the father reported two significant facts: (1) a recent change in his familial structure, when his daughter left home after her marriage; (2) the very close relationship between his children. At that moment, we suggested the hypothesis of self-harm for attention, which led to the presumptive diagnosis of factitious oral ulcers. The lesions were probably produced by self-biting of buccal vestibule and nail-scratching of gingival tissue. These hypotheses were supported by additional reports, describing the boy's habit of introducing his fingers into the mouth, in the same side where the ulcers were observed.

We established the treatment for pain and inflammation control, by the topical application of Gingilone ointment (hydrocortisone acetate 5.0 mg/g, neomycin sulfate 5.0 mg/g, troxerutin 20.0 mg/g, ascorbic acid 0.50 mg/g, and benzocaine 2.0 mg/g, Cosmed Indústria de Cosméticos e Medicamentos S.A., Tamboré, Barueri, São Paulo, Brazil) on the ulcers, in the regime of three times a day for one week. After that, the patient was advised to return periodically until a conclusive diagnosis.

**Figure 5:**
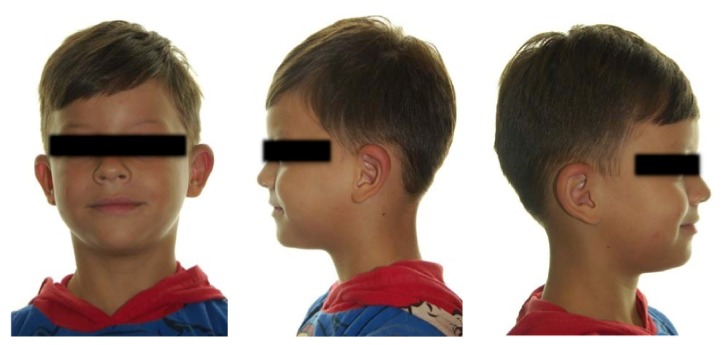
The clinical aspect of the boy at 30-day follow-up appointment.

**Figure 6:**
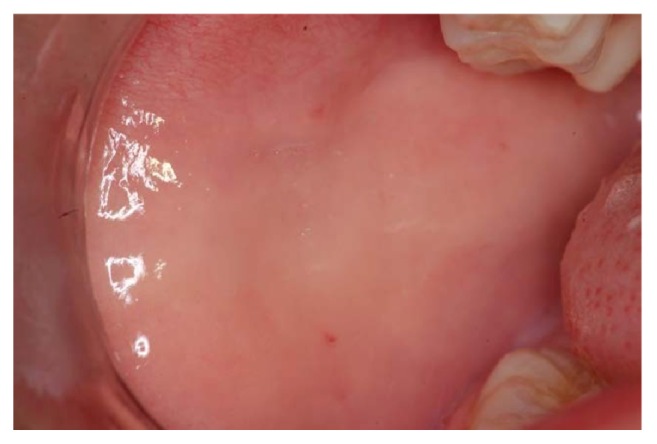
The clinical aspect of health mucosa at 30-day follow-up appointment.

At 7-day follow-up appointment, we observed the swelling regression during the extraoral examination. The intraoral examination revealed the initial healing of the ulcers. At 30-day follow-up appointment, we observed the total remission of the swelling and ulcers on the right side of the mandible (Figures 5 and 6). Thus, the diagnosis of factitious oral ulcers was clinically confirmed by the case resolution.

At this moment, the patient is being periodically monitored to observe the possible recurrence of lesions. We recommended that the parents seek specialized psychological treatment to help the boy with the new family routine.

## 3. Discussion

Self-harm is the aggressive manifestation of the individual with psychological and emotional disturbances [3]. It results in physical damage and pain performed by the individual him/herself [1]. The nonsyndromic factitious ulcers are either intentional or unintentional [2]. The latter is generally related to deleterious habits as lip/thumb sucking, bruxism, nail/other object biting [6].

This present clinical case of factitious oral ulcers was linked directly to the boy's emotional stress caused by a change in his family dynamics. The comprehensive anamnesis provided useful information reported by the father and was of major importance to determine the lesion etiology [4]. Stewart and Kernohan [10] classified the oral factitious ulcer in three types: (A) those not related to a preexisting lesion; (B) secondary lesions caused by chronic habits; (C) lesions of difficult diagnosis due to unknown etiology. This case report showed a type (C) lesion, because of lack of chronic habits or previous lesions, which was confirmed by the emotional and psychological cause. Kwon et al. [11] reported that the differential diagnosis in children is very challenging because they do not provide clear information on their emotional state.

The literature reports the presence of factitious ulcers on oral mucosa and face [7]. Most of the cases is reported in women who injured the oral and perioral structures with the nails, periodically returning to the dentist without knowing to explain how the lesions appear [4, 6, 7]. To the best of our knowledge, the literature lacks reports on the presence of swelling close to the site of the factitious ulcers. Probably, these clinical signs occurred due to an inflammatory response to self-harm. The presence of local infection was discarded because we did not observe important signs as hyperthermia, flushing, and/or face heat.

The clinical treatment of individuals with factitious ulcers is problematic because of the behavior of patients, normally very quiet and shy, reluctant in accepting the self-harm as the cause of the lesion itself [7, 11]. In these cases, the discontinuity of the harmful habits is the target of the treatment planning, which should consider the severity, intensity, and the frequency of the self-harm actions [11, 12]. The multidisciplinary treatment involving dentists, psychologists, and psychiatrist is essential in most severe cases [4, 6, 7, 9, 11].

In clinical failures, the use of mouthguards could be an alternative to restrict the deleterious habits, by the introduction of a customized mouthguard with lingual and labial barriers [13]. This appliance can avoid that the individual interpose the lip, cheek, and tongue between the tooth arches [11, 13], preventing the continuity of self-harm [13]. It is worth noting that the mouthguards do not treat the problem origin. Even the extraction of some or all teeth may be considered as an invasive alternative treatment to avoid persistent self-harm problems in cases without remission [13]. However, this treatment approach is not well accepted by parents.

In this present case, intraoral devices were not necessary to prevent self-harm. We achieved a satisfactory result by a clinical control of lesions with minimal intervention. The instruction of parents and the use of an anti-inflammatory ointment were enough to promote the healing of ulcers and the prevention of their recurrence. Besides, we emphasized the mandatory importance of oral hygiene to prevent secondary infections.

A comprehensive anamnesis associated with clinical examinations is important to the correct diagnosis and proper treatment of factitious oral ulcers. The early diagnosis of lesions and the recognition of their risk factors enable a more conservative clinical approach and the maintenance of the anatomical and physiological health of oral and perioral structures. Due to the lack of available literature on this topic, the establishment of a standardized treatment protocol is difficult. To achieve successful outcomes, the treatments of factitious oral ulcers should be individually tailored for each patient, with focus on a multidisciplinary approach, including psychotherapy and periodic clinical control of possible recurrences.
